# Ectonucleotidase CD38 Demarcates Regulatory, Memory-Like CD8^+^ T Cells with IFN-γ-Mediated Suppressor Activities

**DOI:** 10.1371/journal.pone.0045234

**Published:** 2012-09-17

**Authors:** Rajia Bahri, Annalena Bollinger, Thomas Bollinger, Zane Orinska, Silvia Bulfone-Paus

**Affiliations:** 1 Department of Immunology and Cell Biology, Research Center Borstel, Borstel, Germany; 2 Institut für Medizinische Mikrobiologie und Hygiene, University of Lübeck, Lübeck, Germany; 3 Faculty of Medical and Human Sciences, University of Manchester, Manchester, United Kingdom; National Institute of Allergy and Infectious Diseases, United States of America

## Abstract

Regulatory CD8^+^ T cells are critical for self-tolerance and restricting excessive immune responses. The variety of immune functions they fulfill, the heterogeneity of their phenotype, and the mechanism of action are still poorly understood. Here we describe that regulatory CD8^+^ T cells exhibiting immunosuppressive actions *in vitro* and *in vivo* are recognized as CD38^high^ T cells and present in naive mice. CD38 is a glycosylated membrane protein with ectonucleotidase properties. CD8^+^CD38^high^ (CD44^+^CD122^+^CD62L^high^) lymphocytes suppress CD4^+^ effector T-cell proliferation in an antigen-non specific manner via IFN-γ. While direct cell-to-cell contact is needed for this suppressor activity, it is independent of membrane-bound TGF-β and granzyme B release. IL-15 potentiates the suppressive activity of CD8^+^CD38^high^ T cells and controls their survival and expansion. In humans CD8^+^CD38^high^ T cells inhibit CD4^+^ effector T cell proliferation. *In vivo*, CD8^+^CD38^high^, but not CD8^+^CD38^−^ T cells mitigate murine experimental autoimmune encephalomyelitis (EAE) by reducing the clinical score and delaying disease occurrence. EAE suppression is enhanced by pre-treatment of CD8^+^CD38^high^ T cells with IL-15. These findings add evidence that the expression of ectoenzyme receptor family members positively correlates with suppressor functions and identifies CD8^+^CD38^high^ T cells as potential inhibitors of excessive immune responses.

## Introduction

“Memory-like” regulatory CD8^+^ T cells have witnessed a renaissance as suppressive immunoregulators in pathologies such as autoimmune and infectious diseases and cancer [1], [2], [3]. Impaired numbers and functions of regulatory CD8^+^ T cells (CD8^+^ Tregs) have been defined in various experimental models of autoimmunity. Multiple subsets of CD8^+^ T cells, naturally present in non-immunized mice or induced, have been described to be suppressive [4] and to act through different mechanisms such as secretion of inhibitory cytokines, cytokine-deprivation, granzyme A/B-perforin-induced cytotoxicity and inhibition of dendritic cell functions [5], [6], [7]. For instance, CD8^+^CD28^−^ Tregs, induced *in vitro* by antigen stimulation, express classical regulatory markers such as Foxp3, GITR, CTLA-4, CD25, CD103, CD62L and 4-1BB [8] and produce the immunosuppressive cytokines IL-10 and TGF-β [2], [9]. Their suppressive activities are MHC class I-restricted, contact-dependent and require the presence of antigen presenting cells. Transfer of CD8^+^CD28^−^ Tregs into CD8^+^ T cell-deficient mice significantly suppressed experimental autoimmune encephalomyelitis (EAE) [2].

Antigenic stimulation induces CD8^+^CD103^+^ Tregs in mice and humans. These cells express CD28, lack CD25, Foxp3, GITR, CTLA-4 and LAG-3, and the expression of CD103 is enhanced by TGF-β [10], [11]. CD8^+^CD103^+^ Tregs produce IL-10 and TGF-β and require cell contact and IFN-γ secretion for their inhibitory effect [12]. Furthermore, Qa-1-restricted CD8^+^ Tregs inhibit the development of a lupus-like autoimmune disorder and EAE [13], [14]. Analysis of the phenotype of Qa-1-restricted CD8^+^ Tregs indicated the expression of CD44, CD122 and Ly-49 [15]. Like induced CD8^+^CD25^+^ Tregs [6], [16] also Qa-1-restricted CD8^+^ Tregs require perforin and IFN-γ for their suppressive activity [17].

In mice IL-10 secreting CD8^+^CD122^+^ Tregs were found in the periphery of unimmunized mice and are proposed to be the counterpart of human CD8^+^CXCR3^+^ T cells [18]. Their protective role has been shown in the murine EAE [19] and in a CD4^+^ T cell-induced colitis model [20]. Despite of numerous reports on the existence of CD8^+^ Tregs their functional characteristics, marker profile and mechanisms of action still remain to be defined.

It is increasingly evident that purigenic signaling mediated by ectonucleotidases such as CD39, CD73 and CD38 strongly influences the adaptative immunity [21]. Indeed, the CD39 nucleoside triphosphate diphosphohydrolase together with CD73, ecto-5′-nucleotidase, generate pericellular adenosine from extracellular nucleotides which is required for CD4^+^CD25^+^Foxp3^+^ Tregs-mediated immune suppression [22]. CD38 ectonucleotidase is a multifunctional ectoenzyme, that is able to transform nicotinamide adenosine diphosphate ribose (NAD+) into ADP-ribose (ADPR) and cADP-ribose (cADPR), but also hydrolyses cADPR into ADPR [23], [24]. It is a type II glycosylated membrane protein, ubiquitously expressed on both hematopoietic and non-hematopoietic tissues, which regulates Ca^2+^ mobilization [25]. Recently, CD38 has been found to be associated with regulatory T cell activities. Herein, murine CD45RB^low^ memory CD4^+^ T cells expressing CD38 inhibit anti-CD3-induced T cell proliferation and cytokine secretion [26]. In the absence of CD38, NOD mice develop accelerated autoimmune diabetes, and CD38^−/−^ mice show an impaired regulatory T cell development [27]. CD38 has been shown to compete with ADP-ribosyltransferase 2 (ART2) for NAD, and in this case, CD38 deficiency in Tregs is associated with NAD-induced T cell apoptosis [27], [28]. High expression of CD38 on Foxp3^+^CD4^+^ T cell subpopulations correlates with strongest regulatory properties of CD4^+^ regulatory T cells [29]. In humans, the detection of anti-CD38 autoantibodies is associated with Type I diabetes, chronic autoimmune thyroiditis or Graves' disease [30], [31]. CD38 expression is tightly regulated during lymphocyte development and activation in both humans and mice. While in humans CD38 expression is high on mature thymocytes and activated T cells but low on resting naive T cells [32], CD38 expression in mice is restricted to a αβ TCR^+^CD4^−^CD8^−^ subset of thymocytes but at very low levels on resting T lymphocytes [33]. Interestingly, a subset of activated and memory T lymphocytes expressing CD38 is characterized by low proliferating activity but enhanced IL-2 and IFN-γ production capacity [34].

Unimmunized young mice generate and maintain “memory-like” CD8^+^ T cells of unknown antigen encounter history. However, their immune functions, the heterogeneity of their phenotype and the mechanisms of action are still poorly understood. In this study, we demonstrate that the ectonucleotidase CD38 is highly expressed on memory-like CD8^+^ T cells (CD44^+^CD122^+^CD62L^high^). CD8^+^CD38^high^ T cells exhibited suppressive properties which depend on IFN-γ secretion. We therefore propose that CD8^+^CD38^high^ T cells potentially contribute to T cell homeostasis control.

## Results

### CD38 is Expressed on Memory-like CD8^+^ T Cells

**Figure 1:**
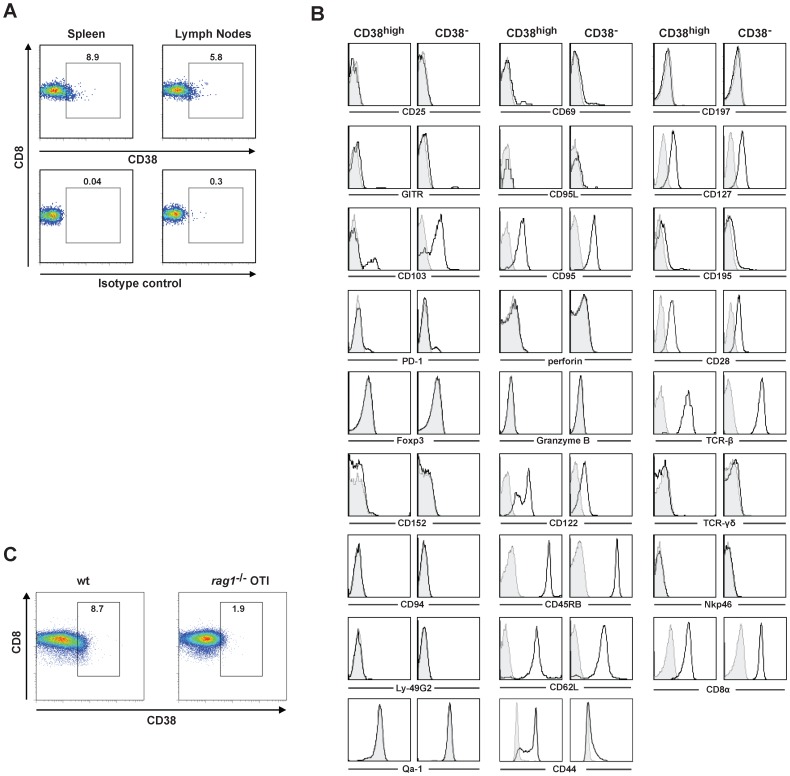
Memory-like CD8^+^ T cells express CD38. Negatively selected CD8^+^ T cells from spleens and lymph nodes were stained with CD8 and CD38 mAbs (1A, upper panel), CD8 and isotype matched control antibodies (1A, lower panel). Intracellular Foxp3, granzyme B, perforin and the indicated cell-surface markers on sorted CD8^+^ T cells from LN were determined by flow cytometry (B). Bold-line histograms show staining with specific antibodies and grey-filled histograms isotype-matched controls. Similar results were observed in at least three independent experiments for each marker (B). Negatively selected splenic CD8^+^ T cells from wt and *rag1* ^−/−^OTI mice were stained with CD8 and CD38 mAbs (C). Numbers represent percentage of cells in the indicated gate. One representative experiment from two is shown.

CD38 has long been considered an activation marker, however high expression of CD38 on CD4^+^ T cell subpopulations has been associated with regulatory properties [26], [29]. More recently, CD38 has been described to be part of the Treg transcriptional signature [35], [36]. Furthermore, it has been shown that CD38-deficient NOD mice display a marked reduction in viable CD8^+^ T cells but a significant increase in β-cell-autoreactive CD8^+^ T cells together with an accelerated autoimmune diabetes [27]. We have analysed the CD38 expression on CD8^+^ T cells from spleens and lymph nodes (LN) (Figure 1A). On average, 8.4%±1.5 of spleen (n = 6) and 5.4%±0.7 of LN (n = 7) CD8^+^ T cells are CD38^high^ (Figure 1A). Resting CD8^+^ T cells are heterogeneously positive for CD38. We have focussed on the CD8^+^CD38^high^ T cell population. We compared the phenotype of these cells to CD8^+^CD38^−^ T cells using a panel of markers for T cell activation and memory function, cytokine receptors and natural killer cell (NK) markers. High levels of CD38 expression correlate with high CD122 and CD44 and low CD103 expression (Figure 1B). However, while 86% of CD8^+^CD38^high^ sorted T cells are CD44 positive only 26% of sorted CD8^+^CD44^high^ are CD38 positive despite a global shift of the population (Figure S1), thus suggesting that CD8^+^CD38^high^ T cells are a unique subpopulation. CD8^+^CD38^high^ T cells from LN do not express conventional regulatory markers such as CD25, Foxp3, GITR, CD152 (CTLA-4) and Qa-1 and are CD95L, granzyme B and perforin negative (Figure 1B). NKp46, Ly-49G2, CD94 and TCR-γδ are expressed on neither CD38^high^ nor CD38^−^CD8^+^ T cells. CD8^+^CD38^high^ and CD8^+^CD38^−^ T cells show no obvious differences in the expression of CD95, CD28, CD45RB, CD195 and PD-1. Both populations were found highly positive for CD62L and CD127 but negative for the activation markers CD197 and CD69. Finally, compared to CD8^+^CD38^−^, CD8^+^CD38^high^ T cells express lower levels of CD8α and TCR-β and show no significant CD103 staining (Figure 1B). Similar results were obtained upon analysis of CD8^+^ T cell subsets in spleens (data not shown). Interestingly the CD8^+^CD38^high^ T cell subset is less represented in spleens of unimmunized *rag1* ^−/−/^TCR-transgenic OTI mice (2.9%±0.75; n = 5) compared to wild-type mice (wt) (8.04%±0.63; n = 5) (p = 0.0079) (Figure 1C), thus indicating that environmental antigens, self-peptides and enteric flora are required for their development *in vivo*. Therefore, we demonstrate that CD38 is expressed on antigen experienced memory-like CD8^+^ T cells of non-immunized mice with the following phenotype CD8^+^CD38^high^CD44^+^CD122^+^CD62L^high^.

### IL-15 Promotes Expansion and Survival of CD8^+^CD38^high^T Cells

**Figure 2:**
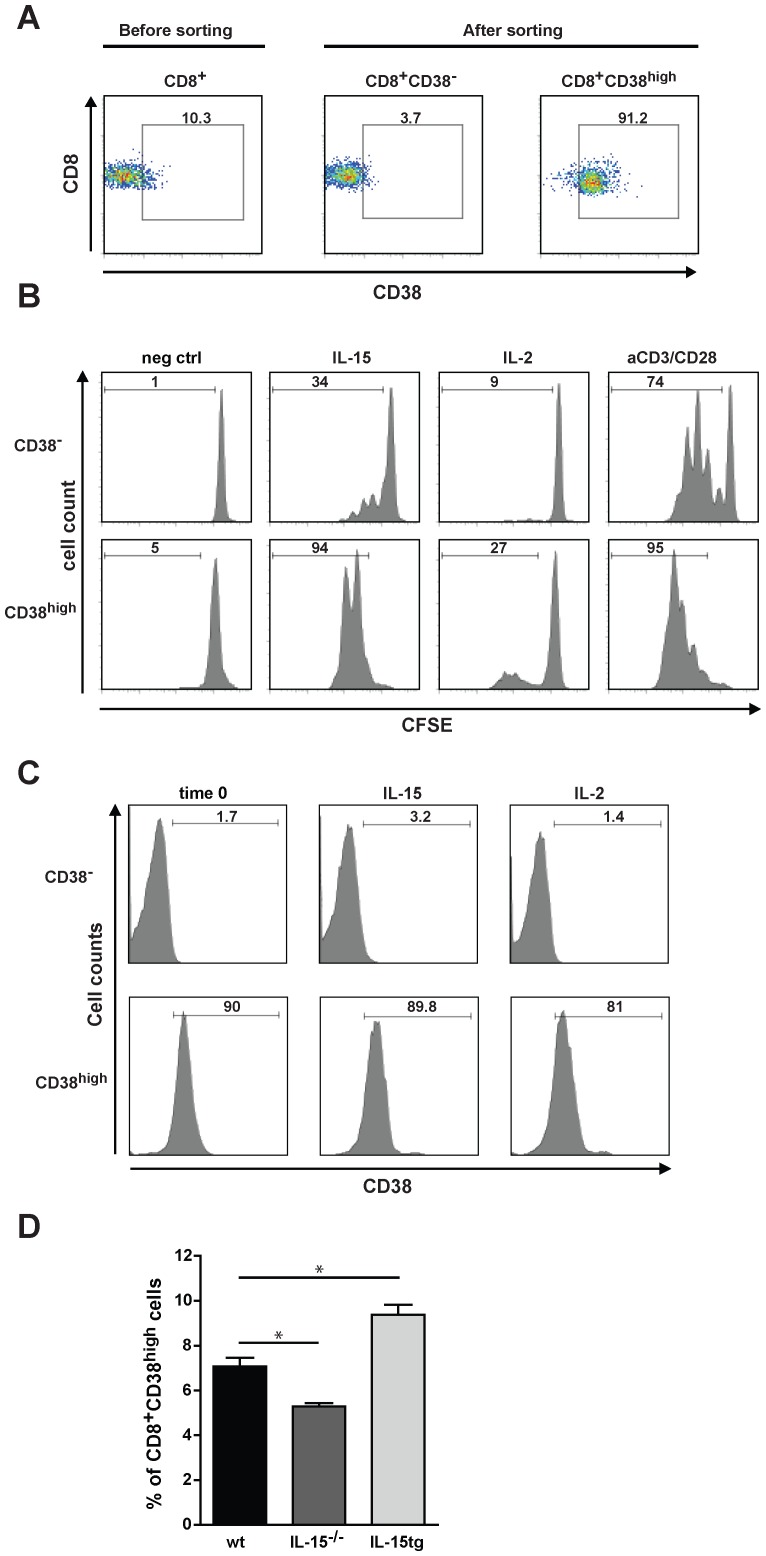
IL-15 promotes expansion and survival of CD8^+^CD38^high^T cells. (A) After a CD8-negative selection by MACS the CD8^+^ T cells isolated from spleens and LN were stained with CD8 and CD38 mAbs and subsequently sorted into CD38^high^ and CD38^−^ T cells by FACS. Flow cytometric analysis of CD8 and CD38 expression on CD8^+^ T cells before and after FACS sorting are shown. (B) Sorted CD8^+^CD38^high^ and CD8^+^CD38^−^ T cells from spleens and LN were treated either with IL-15 (100 ng/mL), IL-2 (100 ng/mL), aCD3/CD28 (1 µg/mL) or left untreated (neg ctrl). After 72 h the proliferation of CD8^+^ T cells was detected by CFSE dilution. Numbers represent the percentage of proliferating cells (B) and CD38 positive cells (C). The results are representative of three independent experiments performed. (D) Spleen cells from wt (n = 4), IL-15^−/−^ (n = 5), and IL-15 tg (n = 5) mice were stained with CD8 and CD38 mAbs and the percentage of CD38^+^CD8^high^ T cells was determined by FACS. Values represent the mean±SEM of one experiment of two performed. *p<0.05 (Mann-Whitney test).

Common gamma chain cytokines contribute to maintenance of memory CD8^+^ T cell homeostasis. To investigate the importance of IL-15 for survival and proliferation of CD8^+^CD38^high^ T cells, the subset was cell-sorted by FACS to a purity of >91% (Figure 2A) and cultured either in medium alone or with IL-15 or IL-2. In the presence of IL-15, more than 90% of CD8^+^CD38^high^ T cells divided at 72h, while only 34% of CD8^+^CD38^−^ T cells were detected to proliferate (Figure 2B). In contrast, supplementation with IL-2 showed a rather low pro-proliferative effect. Both CD8^+^ T cell subsets proliferated to a high extent in response to TCR-engagement (Figure 2B, aCD3/CD28). Both, IL-15 and IL-2 treatment increased the percentage of surviving CD8^+^CD38^high^ and CD8^+^CD38^−^ T cells compared to controls (data not shown). Importantly, neither IL-2 nor IL-15, influenced the expression of CD38 (Figure 2C), indicating that both CD8^+^CD38^high^ and CD8^+^CD38^−^ T cells display a stable phenotype in a 3-day culture.

Since IL-15 modulates the *in vitro* expansion of CD8^+^CD38^high^ T cells and mice over-expressing IL-15 display an increase whereas IL-15^−/−^ mice show a reduction in the total number of CD8^+^ T cells, we have investigated whether this respective deficiency or increment affects the CD8^+^CD38^high^ T cell subset in these transgenic animals. Analysis of spleen cells revealed that CD8^+^CD38^high^ T cells were significantly reduced in IL-15^−/−^ mice, but increased in IL-15tg mice in comparison to wt C57BL/6j controls (Figure 2D). Hence, these results clearly demonstrate that IL-15 mediates the expansion of CD38^high^CD8^+^ T cells *in vitro* and their maintenance *in vivo*.

### CD8^+^CD38^high^ T Cells Suppress CD4^+^ T Cell Proliferation *in vitro*

**Figure 3:**
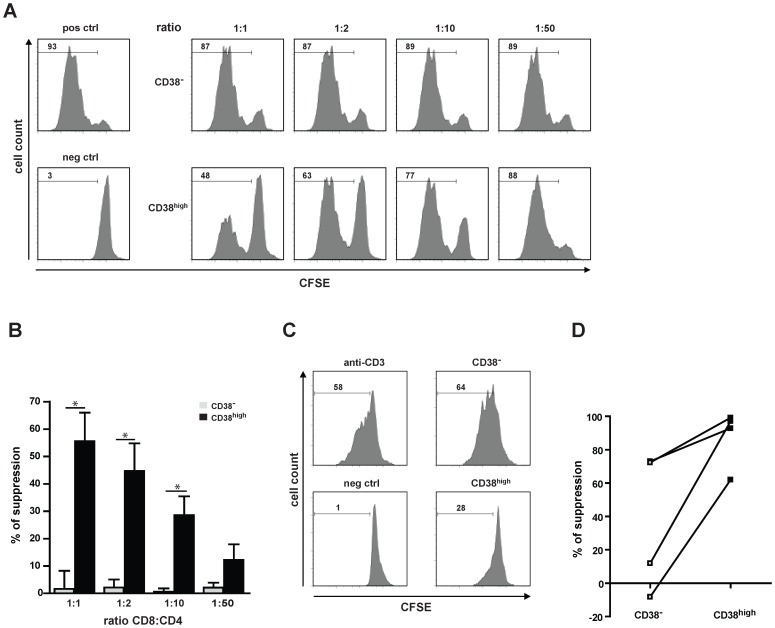
CD8^+^CD38^high^ T cells suppress effector CD4^+^ T cell proliferation. (A) CFSE-labelled CD4^+^ OTII T cells were used as responder and stimulated with OTII^323–339^ peptide in the presence of DCs. Either CD8^+^CD38^high^ (CD38^high^) or CD8^+^CD38^−^ (CD38^−^) T cells were added to the culture at different CD8:CD4 ratios. Histograms show the CFSE dilution of the CD4^+^ OTII T cells after three days of culture (gated on CD4^+^ T cells). Numbers represent the percentage of proliferating cells. Proliferation of CD4^+^ T cells incubated with DC only, or in addition with OTII^323–339^ peptide are shown as a negative (neg ctrl) and positive control (pos ctrl), respectively. One of at least four independent experiments is shown. (B) Quantification of CD8^+^ T cell-mediated suppression of CD4^+^ T cell proliferation. Values represent the mean±SEM of four independent experiments. Percentage of suppression was calculated as: proliferation in the positive control-proliferation in the probe/proliferation in the positive control×100. *p<0.05 (Mann-Whitney test). (C) Suppression of human CD4^+^ T cell proliferation by CD8^+^CD38^+^ T cells. Anti-CD3 stimulated proliferation of CD4^+^ T cells (pos ctrl) in addition of CD8^+^38^high^ or CD8^+^38^−^ T cells, or left unstimulated (neg ctrl) was measured by CFSE dilution after four days of incubation. One out of four independent experiments is shown. (D) Quantification of CD8^+^ T cell-mediated suppression of CD4^+^ T cell proliferation. Values represent percentage of suppression of four independent experiments.

Considering that CD8^+^CD122^+^ T cells are inhibitory *in vitro* [7] and reasoning that CD8^+^ T cells could play an essential role in regulating CD4^+^ T cell responses, we have tested the CD8^+^CD38^high^ T cell-suppressive activity. To this purpose, CD8^+^CD38^high^ and CD8^+^CD38^−^ T cells were co-cultured for 96 h with CFSE-labelled TCR-transgenic CD4^+^CD25^−^ T cells (OTII T cells) and OVA^323–339^ peptide-pulsed DCs (Figure 3A and B). We observed that CD8^+^CD38^high^ T cells isolated from naive wt mice suppressed the antigen-specific proliferative response of CD4^+^CD25^−^ T cells in a dose-dependent manner (ratio CD8:CD4 1:1–1∶50). In contrast, CD8^+^CD38^−^ T cells had a minimal effect on CD4^+^CD25^−^ T cell proliferation (Figure 3A and B). Furthermore, the suppressive activity of CD8^+^CD38^high^ T cells is higher than the one of CD8^+^CD44^high^ T cells (Figure S1) and potentiated by an *in vitro* exposure to IL-15 (Figure S2).

To test whether human CD8^+^CD38^high^ T cells share the above mentioned suppressive activities with their mouse counterparts, CD8^−^ T cells isolated from human PBMCs were labelled with CFSE and stimulated with anti-CD3 antibodies, in the presence of sorted CD8^+^CD38^high^ or CD8^+^CD38^−^ T cells (control). Analysis of effector T cell responses was obtained by gating on CD4^+^ CFSE^+^ T cells (effector CD4 T cells). As shown in Figure 3C, in the presence of CD8^+^CD38^high^, but not of CD8^+^CD38^−^ T cells, the proliferation of CFSE-labelled effector CD4^+^ T cells was inhibited using a CD8^+^CD38^high^ T cell to a CD8^−^PBMC ratio of 1∶3. Figure 3D summarizes the suppressive assay data performed with cells obtained from four different donors. Collectively, these studies demonstrate that both human and mouse CD8^+^CD38^high^ T cells function as suppressor T cells.

### CD8^+^CD38^high^ T Cells Suppressive Activity Correlates with an Increased IFN-γ Secretion and Requires Cell-cell Contact

**Figure 4:**
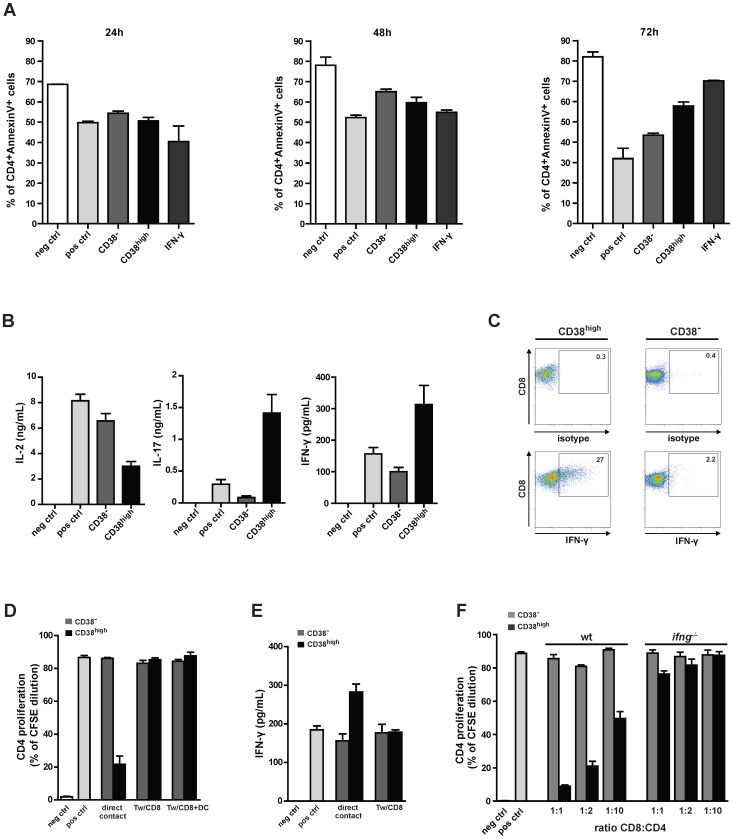
CD8^+^CD38^high^ T cells are suppressive *in vitro*, their activity is dependent on IFN-γ secretion and requires cell-to-cell contact. 1×10^5^ CD4^+^ OTII T cells were stimulated with (pos ctrl) or without (neg ctrl) OTII^323–339^ peptide in the presence of DCs. CD8^+^CD38^high^ (CD38^high^) or CD8^+^CD38^−^ (CD38^−^) T cells were added to the culture at a ratio 1∶1 (1×10^5^ cells) (A-E). (A) Apoptosis of CD4^+^ OTII T cells was measured by annexinV/PI staining. The percentage of CD4^+^ annexinV^+^ T cells is shown at indicated time points of co-culture with CD8^+^ T cells or in the presence of 100 ng/ml of IFN-γ (IFN-γ). The assay was performed in triplicates and shown is the mean±SEM. Results are representative of three independent experiments. (B) Concentrations of IL-2, IL-17 and IFN-γ in supernatants of 72 h co-cultures were measured by ELISA and the mean±SEM of one of three independent experiments is shown. (C) CD4^+^ OTII T cells were stimulated with OTII^323–339^ peptide in the presence of DCs. Either CD8^+^CD38^high^ (CD38^high^) or CD8^+^CD38^−^ (CD38^−^) T cells were added to the culture at a CD8:CD4 ratio of 1 for 48 h. The last 6 h of incubation brefeldin (10 µg/mL), ionomycin (1 µg/mL) and PMA (100 ng/mL) were added to the co-cultures. Cells were stained with CD8 mAbs, fixed and intracellularly stained with IFN-γ mAbs or isotype matched control Abs. CD8^+^ T cells were gated and plots represent the expression of IFN-γ in CD38^high^ or CD38^−^ T cell populations. Numbers represent percentage of cells in the indicated gate from one representative experiment of two performed. (D) CD4^+^ OTII T cells were stimulated without (neg ctrl) or with (pos crtl) OTII^323–339^ peptide in the presence of DCs. Either CD8^+^CD38^high^ (CD38^high^) or CD8^+^CD38^−^ (CD38^−^) T cells were added directly (direct contact) to the culture at a CD8:CD4 ratio of 1. CD4^+^ OTII T cells, DC and OTII^323–339^ peptide were placed in the lower chamber of the transwell, CD38^high^ or CD38^−^ T cells (Tw/CD8) or CD38^high^ or CD38^−^ T cells together with DCs (Tw/CD8+DC) were given into the upper chamber of the transwell (at a CD8:CD4 ratio of 1). (D) Antigen-specific proliferation of CD4^+^ OTII T cells was measured by CFSE dilution after four days of incubation. (E) IFN-γ concentration in the supernatants was measured by ELISA. Assay was performed in triplicates and the mean±SEM of one representative of three independent experiments is shown. (F) CD4^+^ OTII T cells were incubated with DCs with (pos ctrl) or without (neg ctrl) OTII^323–339^ peptide. CD8^+^CD38^high^ (CD38^high^) and CD8^+^CD38^−^ (CD38^−^) T cells sorted from C57Bl/6j wt or *ifn-γ* ^−/−^ mice were added to the culture at the indicated CD8:CD4 ratios. The proliferation of CD4^+^ OTII T cells was measured in triplicates by CFSE dilution after four days of culture. The mean±SEM of one of two independent experiments is shown.

To evaluate, the pro-apoptotic activity of CD8^+^CD38^high^ T cells, Annexin-V and propidium iodide staining of co-cultured CD4^+^CD25- OTII T cells was performed after 24 h, 48 h and 72 h of antigen-specific stimulation (Figure 4A). At 24 h and 48 h no appreciable difference in the percentage of apoptotic CD4^+^ T cells was detected between the co-cultures with CD8^+^CD38^−^ or CD8^+^CD38^high^ T cells. At 72 h, while CD4^+^ T cells from the co-culture with CD8^+^CD38^−^ T cells proliferated comparably to positive controls, CD4^+^ T cells from co-cultures with CD8^+^CD38^high^ T cells were more than 58% Annexin V^+^. These results suggest that the suppressive effect of CD8^+^CD38^high^ T cells is not due to an early induction of apoptosis but the consequence of an absent CD4^+^ T cell proliferation. Interestingly, addition of IFN-γto the co-cultured CD4^+^CD25^−^ OTII T cells had similar effects on proliferation like the addition of CD8^+^CD38^high^ T cells.

To study the underlying mechanism of CD8^+^CD38^high^ T cell-mediated suppression we have investigated whether soluble factor(s) or cell-to-cell contact were involved in this activity. First, the concentration of selected cytokines was measured by ELISA in the supernatant of co-cultures of CD8^+^CD38^high^ T cells with CD4^+^CD25^−^ OTII T/DCs/OVA^323–339^ peptide. After stimulation with OVA^323–339^ peptide, while IL-2 levels were significantly reduced in the co-cultures with CD8^+^CD38^high^ T cells compared to the co-cultures in the presence of CD8^+^CD38^−^ T cells, IL-17 and IFN-γ concentrations were strongly increased (Figure 4B). Unlike the CD8^+^CD122^+^ regulatory T cells previously described in naive mice [7], [37], the CD8^+^CD38^high^ T cell subset did not secret detectable IL-10 and no evident difference in the concentrations of the pro-inflammatory cytokine IL-6 was appreciated in the co-culture supernatants analyzed (data not shown).

To clarify the origin of higher levels of IFN-γ detected in supernatants from the co-culture containing CD8^+^CD38^high^ T cells, we have performed an intracellular IFN-γ staining. As shown in Figure 4C, after 48 h of co-culture 27% of CD8^+^CD38^high^ T cells produced IFN-γ compared to 2.2% of CD8^+^CD38^−^ T cells. Thus, CD8^+^CD38^high^ T cells display higher levels of IFN-γ production. Furthermore, upon addition of CD8^+^CD38^high^ T cells alone or with DCs in the upper chamber of a transwell system, we observed that the presence of a 0.2 µM pore size transwell membrane blocked the suppressive capacity of these cells on CD4^+^ OTII T proliferation (Figure 4D). Addition of DCs to CD8^+^CD38^high^ T cells did not rescue the suppressive activity of these cells (Figure 4D). Moreover, IFN-γ concentrations in supernatants of transwell co-cultures containing CD8^+^CD38^high^ T cells were reduced to control levels (pos ctrl) (Figure 4E). Furthermore, the presence of CD8^+^CD38^high^ T cells, DCs, and CD4^+^ OTII T cells in the upper chamber of the transwell system led to IFN-γ concentrations in the supernatants but had no effect on the CD4^+^ OTII T proliferation in the lower chamber (Figure S3). Finally, the addition of supernatants collected from cultures of CD8^+^CD38^high^ T cells, DCs, and CD4^+^ OTII T cell did not suppress the proliferation of CD4^+^ OTII T cells (Figure S3). Taken together, the suppressive activity of CD8^+^CD38^high^ T cells is dependent on a cell-to-cell contact and correlates with an increase of IFN-γ and IL-17 and a decrease of IL-2 levels in the cell-culture supernatants. We also observed that the exogeneous addition of high concentrations 100 ng/ml of IFN-γ to the cell co-cultures induced suppression of CD4^+^ OTII T cell proliferation (data not shown).

### IFN-γ, but not IL-2 and TGF-β, is Required for the CD8^+^CD38^high^ T Cell-induced Suppression

Next, we have investigated whether CD8^+^CD38^high^ T cells derived from *ifng* ^−/−^ mice are impaired in their ability to suppress proliferation of CD4^+^CD25^−^ OTII T cells. As shown in Figure 4F, CD8^+^CD38^high^ T cells of this mouse strain are indeed unable to exert a suppressive action on CD4^+^ T cell proliferation. Consequently, IFN-γ seems to present a critical mediator for CD8^+^CD38^high^ T cell-mediated suppression (Figure 4F). The results obtained are in agreement with a number of studies indicating the critical role of IFN-γ for CD8^+^ regulatory T cell activities [12], [17], [38].

However in other studies, the suppressive activity of regulatory T cells was shown to be dependent on anti-inflammatory cytokines, e.g. IL-10 and TGF-β, pro-apoptotic factors, e.g. granzyme B, and/or by sequestering activating mediators, e.g. IL-2 [39]. To examine the role of IL-2 and TGF-β in CD8^+^CD38^high^ T cell-mediated suppression, the co-culture of CD8^+^CD38^high^ T cells and CD4^+^CD25^−^ OTII T/DCs/OVA^323–339^ peptide was performed in the presence of exogenous IL-2 or TGF-β blocking antibodies (Figure S4). No differences were observed in the suppressive effect of CD8**^+^**CD38^high^ T cells in the presence of either added IL-2 or TFG-β blocking antibodies. Furthermore, we investigated whether the suppressive activity of CD8^+^CD38^high^ T cells was mediated by the release of granzyme B. As shown in supplementary Figure S4, granzyme B deficient-CD8^+^CD38^high^ T cells showed a suppressive activity on the proliferation of CD4^+^CD25^−^ OTII which was comparable to wt CD8^+^CD38^high^ T cells. These data suggest that the inhibitory effect of CD8**^+^**CD38^high^ T cells is neither primarily due to a shortage of IL-2, as a result of receptor sequestration of the cytokine, nor to the immune suppressive activity of TGF-β nor the release of granzyme B in the culture. In summary, these results indicate that while IL-2, TGF-β and granzyme B seem to be dispensable, IFN-γ is required for the CD8**^+^**CD38^high^ T cell suppressive activity.

### CD8^+^CD38^high^ T Cells Mitigate EAE *in vivo*

**Figure 5:**
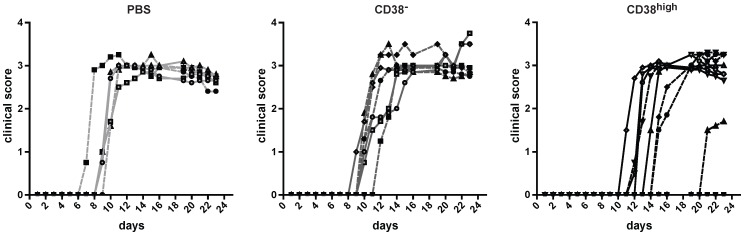
CD8^+^CD38^high^ T cells are suppressive *in vivo*. EAE was induced in C57/BL6j mice. On day 8 mice were injected i.v. either with CD8^+^CD38^high^ cells, CD8^+^CD38^−^ T cells (each 0.75×10^5^ cells/mouse) or with PBS in the negative control. Mice were monitored for disease associated symptoms every day during 23 days. The values represent the clinical scores for each individual mouse of two independent experiments. ctrl (n = 6), CD38^−^ (n = 7), CD38^high^ (n = 9). Similar results were observed in three independent experiments.

Since several studies indicate a regulatory function of CD8^+^ T cells in experimental models of autoimmunity [40], [41], we investigated the suppressive role of CD8^+^CD38^high^ T cells in the EAE model induced by MOG_35–55_ immunization. 0.75 millions of CD8^+^CD38^high^ or CD8^+^CD38^−^ T cells were injected i.v. at day 8 after immunization. As shown in Figure 5, all groups of mice developed disease. However, mice injected with CD8^+^CD38^high^ T cells showed a reduced clinical score, a reduced severity of disease, compared to mice which received CD8^+^CD38^−^ T cells or controls (i.v. PBS). Furthermore, CD8^+^CD38^high^ T cell-treatment significantly delayed both, the onset of clinical symptoms from day 10 (CD8^+^CD38^−^) to day 14 (**p<0.01) and the cumulative clinical score of disease from day 32 (CD8^+^CD38^−^) to day 22 (CD8^+^CD38^high^) (*p<0.05,) (Table 1). However, a minimal delay in the peak of disease was also appreciated upon administration of CD8^+^CD38^−^ T cells compared to PBS. EAE suppression was enhanced by pre-treatment of CD8^+^CD38^high^ T cells with IL-15 (Figure S2). In summary, these results suggest that the inhibitory activity of CD8^+^CD38^high^ T cells affects immune responses *in vivo*.

**Table 1:** Summary of EAE clinical parameters.

Group	Day of onset	Maximal clinical score	Cumulative clinical score
**PBS**	9±1.09	3.07±0.15	35±3.5
**CD38** ^−^	10±0.89	3.2±0.26	32±3.35
**CD38^high^**	14±3.1**	2.9±0.52	22.16±8*

Represents the summary of EAE clinical parameters from two independent experiments calculated over the first 23 days.

* p<0.05,

** p<0.01 (Mann-Whitney test between CD38^−^ and CD38^high^ group).

## Discussion

Here, we establish that CD8^+^CD44^+^CD122^+^CD62L^high^ T cells which are CD38^high^ represent a distinct regulatory T cell population. In non-immunized mice we demonstrate the existence of CD8^+^CD38^high^ T cells that exibit suppressor functions *in vivo* and *in vitro*. Suppression is exerted through contact-dependent inhibition of Teff-proliferation, decrease of IL-2 along with an increased IFN-γ secretion. IL-15 potentiates the suppressive activity of CD8^+^CD38^high^ T cells, and controls their survival and expansion. Finally, in a murine EAE model, CD8^+^CD38^high^ T cells reduce the clinical score and delay disease occurrence.

“Memory-like” T cells of unknown antigen encounter-history represent 10–20% of T cells in young, normal and unmanipulated mice. While these cells have recently become a focus of interest in basic and clinical immunology, the immune functions they fulfill and their characteristics are still little understood. Environmental antigens, self-peptides and enteric flora are assumed to be the stimuli which convert naive into T cells with a memory phenotype [42], [43]. Here we characterized regulatory properties of CD8^+^ T cells with memory-like phenotype demarcated by the expression of CD38. Since these cells are significantly reduced in spleens of unimmunized *rag1* ^−/−/^TCR-transgenic OTI mice, their origin still needs to be defined but an antigen-encounter seems to be required for their memory phenotype acquisition. Monitoring of the induction of CD8^+^CD38^ high^ T cells upon pathogenic infection and their functionality in the future should deliver more in depth knowledge on the origin and suppressive potential of these cells.

CD8^+^CD38^high^ T cells suppress the proliferation of CD4^+^CD25^−^ effector T cells. Recently, the expression of CD38 on CD4^+^CD25^+^ Tregs cells has been correlated with a superior suppression potential. Namely, the CD4^+^CD25^+^CD38^high^ Tregs showed a greater strength in suppression than the CD4^+^CD25^+^CD38^low^ Tregs suggesting that CD38 marks Tregs with high suppressive activity [29]. This evidence strongly supports our findings on the role of CD38 as a marker for suppression in T cells. CD38 is both, a cell surface enzyme and a receptor. The contribution of CD38 to the regulation of T cell activation has been shown by the use of agonist anti-CD38 antibodies that mimic the natural ligand CD31 and lead to the induction of T cell proliferation, protein phosphorylation and cytokine production. The colocalization of CD38 and CD3 to the lipid rafts was shown to be essential for T cell signaling [44], [45]. Whether an active engagement of CD38 is also necessary for the suppressive activity of CD4^+^ and/or CD8^+^ T cells as described above for the control of T cell activation is yet to be defined.

Several mechanisms have been implicated to explain the inhibitory activity of CD8^+^ Treg cells, including direct lysis of target cells and secretion of immunosuppressive cytokines. Compared to a previous study that showed that CD8^+^ Tregs are suppressive via the expression of e.g. IL-10, CTLA-4 or TGF-β [46] we could not confirm the involvement of these mediators in the suppressive activity of CD8^+^CD38^high^ T cells. Furthermore, we showed that CD8^+^CD38^high^ T cells did not induce CD4^+^ T cells apoptosis at early time points and that their suppressive activity was also granzyme B and perforin independent but required cell-to-cell contact and IFN-γ secretion. We have also shown that CD8^+^CD38^high^ T cells from IFN-γ deficient mice were not able to suppress effector T cell proliferation and we found higher concentrations of IFN-γ in the supernatants of co-cultures with CD8^+^CD38^high^ T cells. Moreover, we demonstrated a contact-dependency of CD8^+^CD38^high^ T cells with CD4 effector T cells for the production of IFN-γ. The key role of IFN-γ for suppression is in line with a recent report in which expanded CD8^+^ Treg cells produced IFN-γ and required cell-to-cell contact to suppress CD4^+^ T cell proliferation. However, this is accompained by a concomitant IL-10 production that we failed to detect in our experimental settings [47]. Similar to our data, other studies show that IL-10 did not contribute to Qa-1-restricted suppression by CD122^+^CD44^+^CD8^+^ Treg [13].

In our study, the subpopulation of CD8^+^ CD38^high^ T cells isolated from naive mice by using CD8^+^ and CD38^high^ markers displays a memory-like phenotype which considerably overlaps with the previously described CD8^+^CD122^+^ Tregs [15]. Murine regulatory CD8^+^CD122^+^ T cells and CD8^+^CD38^high^ T cells display a memory phenotype (CD44^+^CD62L^+^). One could argue that CD8^+^CD38^high^ T cells which are Foxp3^−^ may represent a subpopulation of CD8^+^CD122^+^ T cells. The CD122 receptor is considered as both, a regulatory and a memory marker in CD8^+^ T cells, and alone it is not sufficient to define Treg subsets [48]. Nevertheless, the CD8^+^CD122^+^ cell population clearly contains T cells with regulatory properties [37]. CD8^+^CD38^high^ T cells belong to the CD8^+^CD122^+^CD44^+^ regulatory population, but they differ since they do not express PD-1 and Ly-49G2 on their surface. Because CD38 defines CD25^+^CD4^+^ Tregs with high suppressive activity [29], and the CD38 defiency inhibits Treg development [49], our study suggests that CD38 is a marker which further narrows the highly suppressive CD122^+^CD44^+^CD8^+^ Treg cell population.

The development of both, CD8^+^CD122^+^ and CD8^+^CD38^high^ T cells remains unclear. Despite the fact that they do exist naturally in the periphery of non-immunized mice and healthy humans, the thymic origin of these cells has not been proven, yet. Some studies suggest that CD8^+^CD122^+^ T cells may develop in the periphery in response to the contact with autoantigens [50], [51]. Tsai and colleagues support the hypothesis that many regulatory T cell subsets share a memory-like phenotype resulting from a previous antigenic stimulation and are needed to generate a negative feedback regulatory loop that would be able to inhibit the progression of autoimmunity or to promote the chronicity of infection or the progression of cancer [52]. Our study further supports this hypothesis suggesting that CD8^+^CD38^high^ T cells are a subset of memory phenotype T cells in naive mice with suppressive functions which expand and potentiate their inhibitory activity in an IL-15 rich microenvironment. Thus, these cells might be clinically potentially important by regulating self-tolerance and restricting excessive immune responses.

In summary, we have described CD8^+^CD38^high^ T cells as potent immunosuppressive cells *in vitro* and *in vivo*, which constitute attractive potential therapeutic targets for the treatment of immune responses. Finally, we suggest that CD8^+^CD38^high^ T cells may function as regulatory cells that together with innate lymphoid cells operate in controlling immune homeostasis and attenuating inflammatory damage.

## Materials and Methods

### Mice

All mice were on a C57BL/6 background. C57BL/6j wt mice, IL-15^−/−^ [49], IL-15 tg C57BL/6 strain B1 (transgenic for IL-15) [53], C57BL/6 OTII mice, whose transgenic CD4-TCRs specifically recognize the OVA (323–339) epitope were provided from Charles River, C57BL/6 *rag1^−/−^*OTI from Harlan Laboratories, *ifng* ^−/−^ (B6.129S7-*Ifng* ^tm1Ts/J^) from Jackson Laboratory, *gzb* ^−/−^ mice were obtained from the Bernhard Nocht Institute for Tropical Medicine of Hamburg. Mice were maintained at the Research Center Borstel Animal Facilities. Female mice were used between 8–12 weeks of age if not indicated otherwise and were maintained in specific pathogen-free conditions. All *in vivo* experiments were performed in compliance with national and institutional guidelines.

### Cell Isolation and Purification

CD8^+^ T cells were purified from spleens and LN (inguinal, axillar and mesenteric) of C57Bl/6j mice by immunomagnetic depletion using FITC labelled antibodies against CD4, CD11b, CD11c, B220, DX5 (all from BD Pharmingen), F4/80 antibodies (Serotec) and FITC microbeads (Miltenyi Biotec) and AutoMACS® (Miltenyi Biotec). Negative purified CD8^+^ cells (>94% pure) were stained with anti-CD8 and anti-CD38 antibodies, and the CD8^+^CD38^high^ cells and CD8^+^CD38^−^ cells were isolated by using FACSAria cell sorter (BD Biosciences). The final cell purity was >91% for CD38^high^ and >96% for CD38^−^. CD4^+^CD25^−^ T cells were purified from lymph nodes of OTII mice by immunomagnetic depletion using labelled αCD8a, αCD11b, αCD11c, αB220, αDX5, αCD25 (all from BD Pharmingen), αF4/80 antibodies (Serotec) and microbeads (Miltenyi Biotec) and AutoMACS® (Miltenyi Biotec).

For the generation of bone marrow derived dendritic cells (BMDC), C57BL/6j wt mice bone marrow cells were cultivated in RPMI-1640 medium supplemented with 10% heat inactivated FCS, 50 µM 2-mercaptoethanol, 2 mM L-glutamine, 100 U/ml penicillin, and 100 µg/ml streptomycin (all from Gibco ) in presence of 20 ng/ml murine recombinant GM-CSF. After one week of culture, CD11c^+^ cells represented >98% of the total cells.

### Cell Culture

Cells were cultured in RPMI-1640 supplemented with 10% heat inactivated FCS, 50 µM 2-mercaptoethanol, 2 mM L-glutamine, 100 U/ml penicillin, and 100 µg/ml streptomycin (all from Gibco). In some conditions, CD8^+^ cells were cultured at 1×10^6^ cells/ml with 100 ng/mL of IL-15 (recombinant human from R&D) or IL-2 (Human IL-2 from Biotest Pharma) for 48 h or 72 h.

### Immunophenotypic FACS Analysis

Cells were resuspended in FACS-buffer (2% newborn calf serum, 0.1% NaN3, 2 mM EDTA in PBS), stained with mAb against CD8α (53-6.7), CD4 (RM4-5), (DX5), CD25 (7D4), CD28 (37.51), CD38 (90), CD44 (IM7), CD45RB (16A), CD62L (MEL-14), CD69 (H1.2F3), CD95 (Jo2), CD95L (Kay-10), CD103 (M290), CD122 (TM-β1), CD152 (UC10-4F10-11), CD195 (C34-3448), GITR (DTA-1), TCR-β (H57-597), Qa-1 (6A8.6F10.1A6) (all from BD PharMingen), CD197 (4B12), CD94 (18d3), CD127 (A7R34), Ly-49G2 (4D11), NKp46 (29A1.4), PD-1 (J43), TCR-γδ (eBioGL3) (eBioscience), for 20 min at 4°C and subsequently washed with 1 µg/ml propidium iodide (Sigma-Aldrich) in FACS buffer. Cells were analyzed by using a FACSCalibur flow cytometer (BD Biosciences). Intracellular FACS staining was performed according to a standard protocol from eBioscience after a surface staining. Intracellular staining after fixation and permeabilization was performed by incubation with mAb against Foxp3 (FJK-16 s), granzyme B (16G6), perforin (OMAK-D) or IFN-γ (XMG1.2) all from eBioscience.

### Proliferation Assay

1×10^5^ CD4^+^CD25^−^OTII TCR tg Teff cells were used as responder cells and stimulated with 0.1 µM OTII peptide (OVA^323–339^) in presence of BMDCs (1×10^4^). For the proliferation assay, CD4^+^CD25^−^ OTII cells were labeled with 3 µM CFSE (Vybrant CFDA SE Cell Tracker Kit, Molecular Probes) for 6 min at room temperature. The reaction was stopped by addition of FCS, cells were washed and proliferation was measured by FACS 3–4 days later. In order to suppress proliferation of Teff, purified CD8^+^CD38^high^ or CD8^+^CD38^−^ T cells were added in a ratio 1∶1, 1∶2, 1∶10 and 1∶50 (Treg:Teff). Cells were seeded into 96-well, round-bottom microtiter plates (Nunc) in a total volume of 200 µl of RPMI-1640 supplemented with 10% heat inactivated FCS, 50 µM 2-mercaptoethanol, 2 mM L-glutamine, 100 U/ml penicillin, and 100 µg/ml streptomycin (all from Gibco). Each condition was set in triplicates and maintained for 3–4 days at 37°C and 5% CO_2_. CD4^+^ cells were analyzed for CFSE proliferation. To prevent cell contact between Teff and CD8^+^CD38^high^ or CD8^+^CD38^−^ T cells, anopore transwell membranes with a pore size of 0.2 µm (Nunc) were used. For IFN-γ intracellular staining, the last 6 h of a 48 h incubation brefeldin (10 µg/mL from Sigma), ionomycin (1-µ g/mL from Sigma) and PMA (100 ng/mL from Sigma) were added to the co-culture.

### Suppression Assay with Human CD8^+^CD38^high^ T Cells

All human subjects gave written consent and the protocol was approved by the ethic commission of the University of Lübeck. Whole blood was drawn from a forearm catheter and PBMC from whole blood were isolated using Vacutainer® CPTT (BD Bioscience) following the manufacturer’s instructions. Plasma was collected and heat inactivated for 30 min at 56°C and centrifuged at 4,500 ×g for 10 min. Negative selection of CD8^+^ T cells was performed by applying the AutoMacs® (Miltenyi Biotec) using a CD8^+^ T Cell Isolation Kit (Miltenyi Biotec). The remaining cells PBMC CD8^−^ (CD8^−^) were stained with CFSE (Vybrant CFDA SE cell tracer kit, Invitrogen) following the manufacturer’s instructions and kept at 37°C until assayed. The isolated CD8^+^ T cells were separated in CD38^+^ high (CD38^high^) and CD38^−^ cells by FACS sorting. Purities were on average 95% for CD8^+^CD38^high^ and 98% for CD8^+^CD38^−^ T cells.

For the functional analysis of CD8^+^CD38^high^ T cells and CD8^+^CD38^−^ T cells, 1.5×10^5^ CD8^−^ CFSE^+^ cells and either 5×10^4^ CD8^+^CD38^−^ T cells or 5×10^4^ CD8^+^CD38^high^ T cells were incubated in 200 µl X-Vivo 15 medium (Cambrex) supplemented with 1% inactivated, autologous plasma and stimulated with 0.5 µg/ml anti-CD3 antibodies (clone Okt3, eBioscience) for 62 h. Negative controls were cultured without anti-CD3 antibodies and as positive control we stimulated 1.5×10^5^ CD8^−^CFSE^+^ T cells with 0.5 µg/ml anti-CD3. T cell proliferation was analysed by gating on the CD4^+^ T cells and measuring CFSE dilution using flow cytometry.

### Apoptosis Assay

Cells were resuspended in FACS-buffer (2% newborn calf serum, 0.1% NaN3, 2 mM EDTA in PBS), stained with fluorochrome-conjugated mAbs and then the cells were evaluated for apoptosis with the annexin V-FITC apoptosis detection kit (Bender MedSystems GmbH).

### Analysis of Cytokine and Antibody Production

Cytokine measurements in culture supernatants were performed using the enzyme-linked immunosorbent assay (ELISA) for IL-2, IL-17, IFN-γ, IL-6 and IL-10 (R&D Systems).

### EAE Model

Mice were injected s.c. in both flanks with a total of 200 µg of MOG35–55 peptide (MEVGWYRSPFSRVVHLYRNGK) (NeoS) dissolved in PBS emulsified in an equal volume of CFA (Difco) supplemented with 4 mg/ml *Mycobacterium tuberculosis* H37RA (Difco), and injected twice i.v. with 300 ng of pertussis toxin (Calbiochem) administered on the day of immunization and 48 h later. At day 8 after immunization, 0.75×10^6^ of CD8^+^CD38^high^ or CD8^+^CD38^−^ cells were injected i.v. per mice. For the control PBS was injected. Mice were monitored daily and the clinical signs of EAE were graded on the following scale 0: no disease symptoms, 1: limp tail, 2: disability of hind limbs, 3: complete hind paralysis, 4: complete hind paralysis and front limb disability, 5: moribund or dead.

### Statistical Analysis

Results are presented as the mean±SEM from pooled data or as representative data of independent experiments, all of which gave comparable results. Mann-Whitney test was used to determine statistical significant differences *p<0.05, **p<0.01, ***p<0.001.

### Ethics Statement

This study was carried out in strict accordance with the recommendations in the guide for the care and use of Laboratory Animals. Protocols were reviewed and approved by the “Ministerium für Landwirtschaft, Umwelt und ländliche Räume des Landes Schleswig-Holstein” (# V 312-72241.123-3 (38-3/09)). Experiments using human blood cells were approved by the ethics committee of the University of Lübeck.

## Supporting Information

Figure S1 **Comparative analysis between CD8^+^CD44^high^ and CD8^+^CD38^high^ T cells.** (A) Negatively selected CD8^+^ T cells from spleens and LN were stained with CD38 and CD44 mAbs (upper panel). Sorted CD8^+^CD38^high^ and CD8^+^CD44^high^ T cells (lower panel) were stained with CD44 and CD38 mAbs, respectively (bold-lines). Grey-filled histograms show the staining with isotype-matched control Abs. Numbers represent percentage of cells in the indicated gate. (B) CFSE-labeled CD4^+^ OTII T cells were used as responder and stimulated with OTII^323–339^ peptide in the presence of DCs. Either CD8^+^CD38^high^ (CD38^high^) and CD8^+^CD38^−^ (CD38^−^) or CD8^+^CD44^high^ (CD44^high^) and CD8^+^CD44^−^ (CD44^−^) T cells were added to the culture at a CD8:CD4 ratio of 1. CD8^+^ T cell mediated-suppression of CD4^+^ OTII T cell proliferation was measured and the percentage of suppression was calculated as: proliferation in the positive control-proliferation in the probe/proliferation in the positive control×100. The mean±SEM of one representative experiment of two performed is shown.
(TIF)

Figure S2 **IL-15 potentiates the suppressive activity of CD8^+^CD38^high^ T cells.** CD4^+^ OTII T cells were stimulated with OTII^323–339^ peptide in presence of DCs. Different ratios of CD8^+^CD38^high^ (CD38^high^) or CD8^+^CD38^−^ (CD38^−^) T cells, pretreated for 48h with IL-15 (100 ng/mL), were added to the culture and proliferation of CD4^+^ OTII T cells was measured. Histograms show the CFSE dilution of the CD4^+^ OTII T cells after three to four days of culture (gated on CD4^+^ T cells) (A). Numbers represent the percentage of proliferating cells. Pos ctrl represents the proliferation of CD4^+^ T cells incubated with DC and OTII^323–339^ peptide (without addition of CD8^+^ T cells), neg ctrl represents the proliferation of CD4^+^ T cells incubated only with DCs. One of at least four independent experiments is shown. (B) Quantification of suppression of CD4^+^ T cell proliferation when IL-15 pretreated CD8^+^ T cells were added to the cultures. Values represent the mean±SEM of four independent experiments. (C) C57/BL6j wt mice were injected s.c. in both flanks with a total of 200 µg of MOG^35–55^ peptide with 4 mg/ml *Mycobacterium tuberculosis*, and injected twice with 300 ng of pertussis toxin administered the day of immunization and 48 h later. On day 8 the mice were injected i.v. either with IL-15 pretreated CD8^+^CD38^high^ T cells (CD38^high^ IL-15- treated), CD8^+^CD38^−^ T cells (CD38^−^ IL-15-treated) (each 0.75×10^5^ cells/mouse) or PBS. Mice were monitored for disease associated symptoms every day for 15 days. The values represent the clinical score shown as mean for each group of two experiments. ctrl (n = 5), CD38^−^ (n = 8), CD38^high^ (n = 8). **p<0.01 (Mann-Whitney test between the CD38^−^ and CD38^high^ treated group).
(TIF)

Figure S3 **CD8^+^CD38^high^ T cell mediated-suppression is dependent on cell-to-cell contact.** (A-B) CD4^+^ OTII T cells and DCs with (pos ctrl) or without (neg ctrl) OTII^323–339^ peptide were placed in the lower chamber of the 0.4µm transwell (Corning). Medium (medium) or indicated CD8^+^ T cells at a CD8:CD4 ratio of 1, CD4^+^ OTII T cells, DC and OTII^323–339^ peptide (CD8+DC+CD4) were given into the upper chamber of the transwell. (A) Antigen-specific proliferation of CD4^+^ OTII T cells from the lower chamber was measured by CFSE dilution after four days. (B) IFN-γ concentration in supernatants was measured by ELISA. Shown is the mean±SEM. (C) CD4^+^ OTII T cells were stimulated with the OTII^323–339^ peptide in the presence of DCs (pos ctrl). Supernatants collected from cultures with either CD4^+^ T cells and DCs (neg ctrl), CD4^+^ T cells, DCs and OTII^323–339^ peptide (pos ctrl) or CD4^+^ T cells, DCs, OTII^323–339^ peptide and either CD8^+^CD38^−^ (CD38^−^) or CD8^+^CD38^high^ (CD38^high^) T cells were added to the above described co-cultures at a dilution 1∶2. CD4^+^ OTII T cell proliferation was measured by CFSE dilution after three days of incubation. Shown is the mean±SEM of one representative experiment from two performed.
(TIF)

Figure S4 **Mechanisms of CD8^+^CD38^high^ T cell-mediated suppression of effector CD4** ^+^ **T cell proliferation.** CD4^+^ OTII T cells were used as responder and stimulated with OTII^323–339^ peptide in the presence of DCs. Different ratios of CD8^+^CD38^−^ (CD38^−^) or CD8^+^CD38^high^ (CD38^high^) T cells were added to the culture and proliferation of CD4^+^ OTII T cells was measured. Histograms show the CFSE dilution of the CD4^+^ OTII T cells after three to four days of culture (gated on CD4^+^ T cells). Numbers represent the percentage of proliferating cells. Pos ctrl represents the proliferation of CD4^+^ T cells incubated with DC and OTII^323–339^ peptide (without addition of CD8^+^ T cells). neg ctrl represents the proliferation of CD4^+^ OTII T cells incubated with DCs only (A-C). (A) IL-2 (100 ng/mL), (B) TGF-β Abs or isotype control Abs were added to the co-cultures or left untreated. (C) CD8^+^CD38^high^ and CD8^+^CD38^−^ T cells were sorted from C57Bl/6j wt or *grzb^−/−^* mice. The proliferation of CD4^+^ OTII T cells was measured by CFSE dilution after four days of culture. Shown is the mean±SEM of one of two independent experiments performed.
(TIF)
