# Supplementary figures and images for: Ectonucleotidase CD38 Demarcates Regulatory, Memory-Like CD8+ T Cells with IFN-γ-Mediated Suppressor Activities
Source: PLoS One. 2012 Sep 17;7(9):e45234. doi: 10.1371/journal.pone.0045234 (PMC3444472; doi:10.1371/journal.pone.0045234)

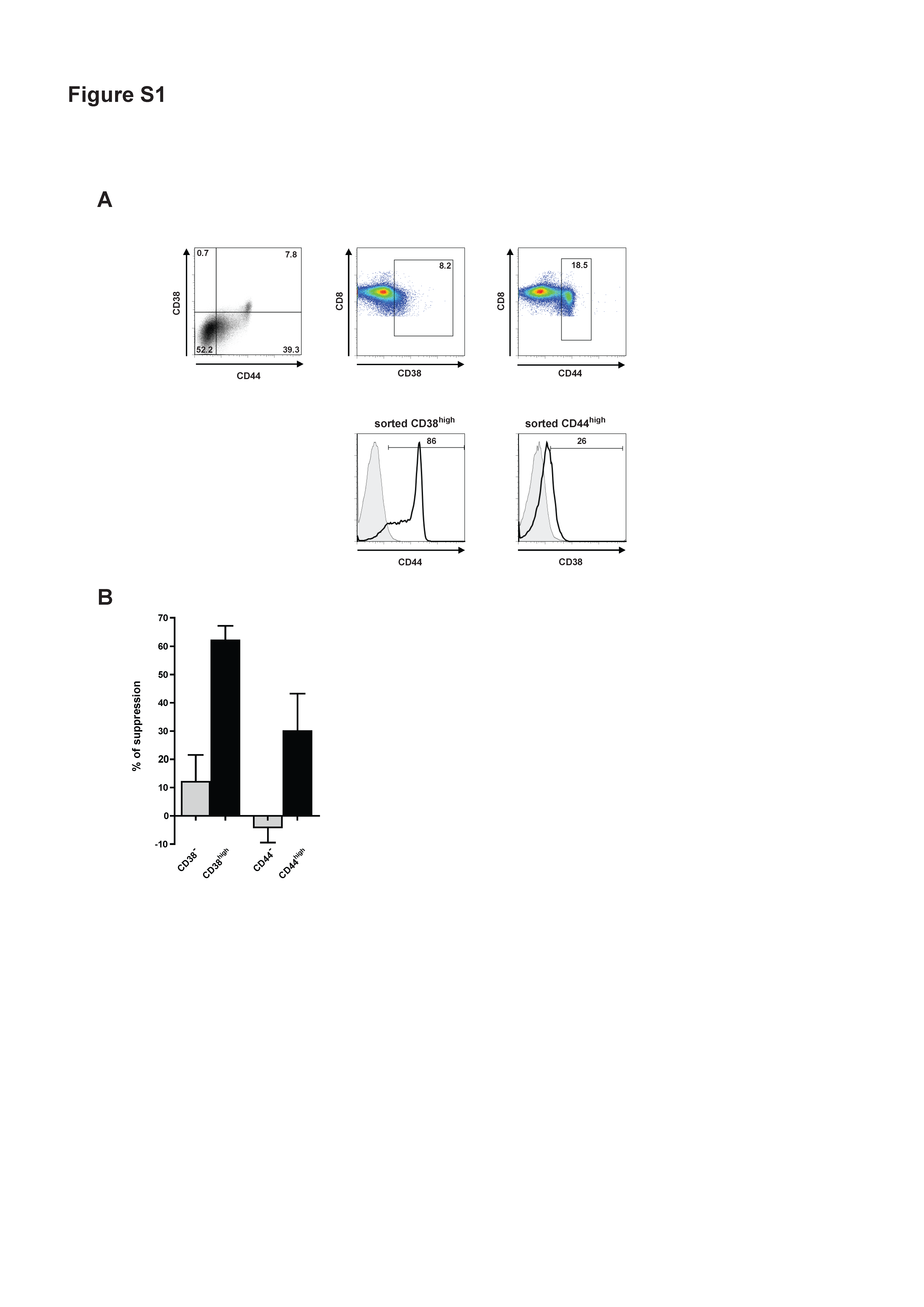

Supplement: Figure S1 — Comparative analysis between CD8+CD44high and CD8+CD38high T cells. (A) Negatively selected CD8+ T cells from spleens and LN were stained with CD38 and CD44 mAbs (upper panel). Sorted CD8+CD38high and CD8+CD44high T cells (lower panel) were stained with CD44 and CD38 mAbs, respectively (bold-lines). Grey-filled histograms show the staining with isotype-matched control Abs. Numbers represent percentage of cells in the indicated gate. (B) CFSE-labeled CD4+ OTII T cells were used as responder and stimulated with OTII323–339 peptide in the presence of DCs. Either CD8+CD38high (CD38high) and CD8+CD38− (CD38−) or CD8+CD44high (CD44high) and CD8+CD44− (CD44−) T cells were added to the culture at a CD8:CD4 ratio of 1. CD8+ T cell mediated-suppression of CD4+ OTII T cell proliferation was measured and the percentage of suppression was calculated as: proliferation in the positive control-proliferation in the probe/proliferation in the positive control×100. The mean±SEM of one representative experiment of two performed is shown. (TIF) [file pone.0045234.s001.tif]

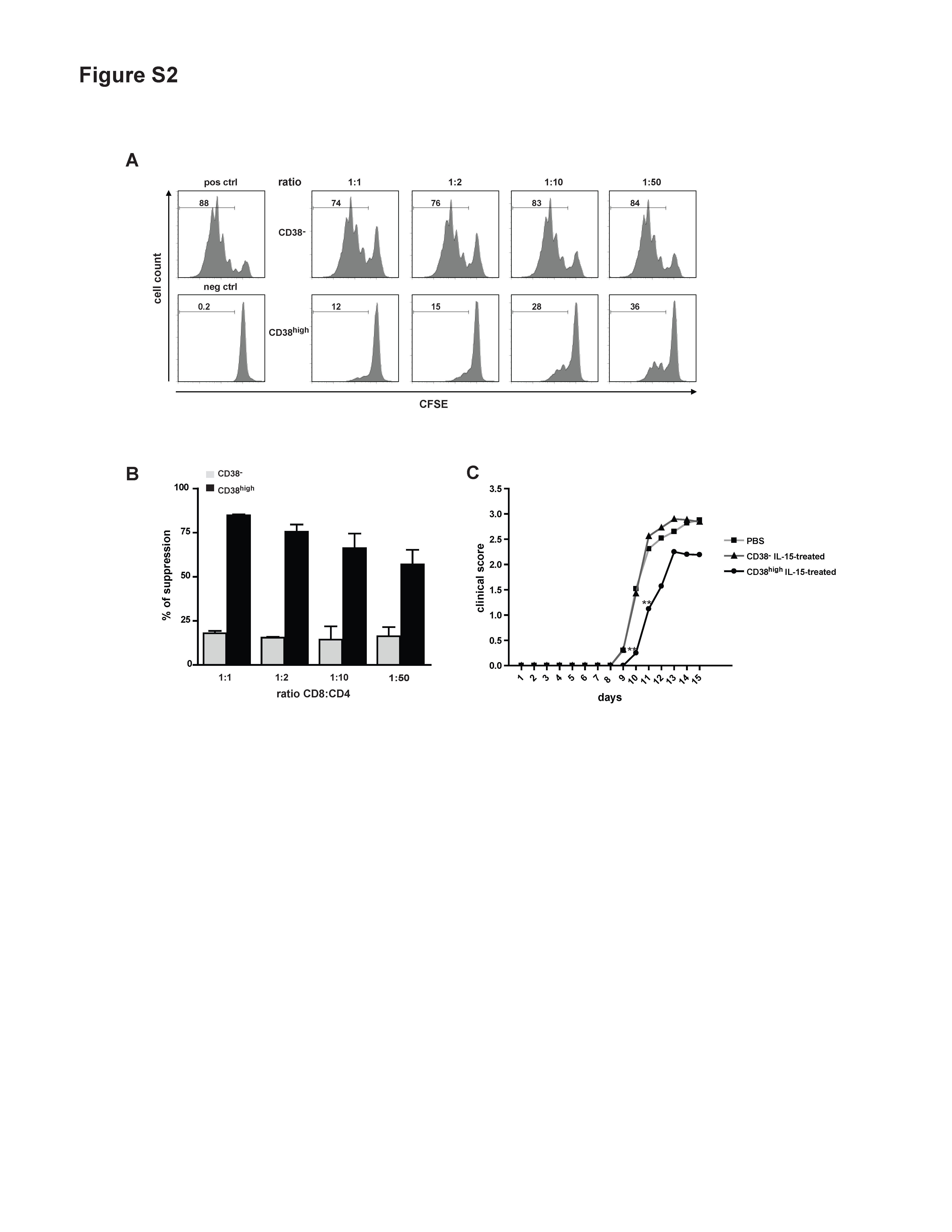

Supplement: Figure S2 — IL-15 potentiates the suppressive activity of CD8+CD38high T cells. CD4+ OTII T cells were stimulated with OTII323–339 peptide in presence of DCs. Different ratios of CD8+CD38high (CD38high) or CD8+CD38− (CD38−) T cells, pretreated for 48h with IL-15 (100 ng/mL), were added to the culture and proliferation of CD4+ OTII T cells was measured. Histograms show the CFSE dilution of the CD4+ OTII T cells after three to four days of culture (gated on CD4+ T cells) (A). Numbers represent the percentage of proliferating cells. Pos ctrl represents the proliferation of CD4+ T cells incubated with DC and OTII323–339 peptide (without addition of CD8+ T cells), neg ctrl represents the proliferation of CD4+ T cells incubated only with DCs. One of at least four independent experiments is shown. (B) Quantification of suppression of CD4+ T cell proliferation when IL-15 pretreated CD8+ T cells were added to the cultures. Values represent the mean±SEM of four independent experiments. (C) C57/BL6j wt mice were injected s.c. in both flanks with a total of 200 µg of MOG35–55 peptide with 4 mg/ml Mycobacterium tuberculosis, and injected twice with 300 ng of pertussis toxin administered the day of immunization and 48 h later. On day 8 the mice were injected i.v. either with IL-15 pretreated CD8+CD38high T cells (CD38high IL-15- treated), CD8+CD38− T cells (CD38− IL-15-treated) (each 0.75×10^5^ cells/mouse) or PBS. Mice were monitored for disease associated symptoms every day for 15 days. The values represent the clinical score shown as mean for each group of two experiments. ctrl (n = 5), CD38− (n = 8), CD38high (n = 8). **p<0.01 (Mann-Whitney test between the CD38− and CD38high treated group). (TIF) [file pone.0045234.s002.tif]

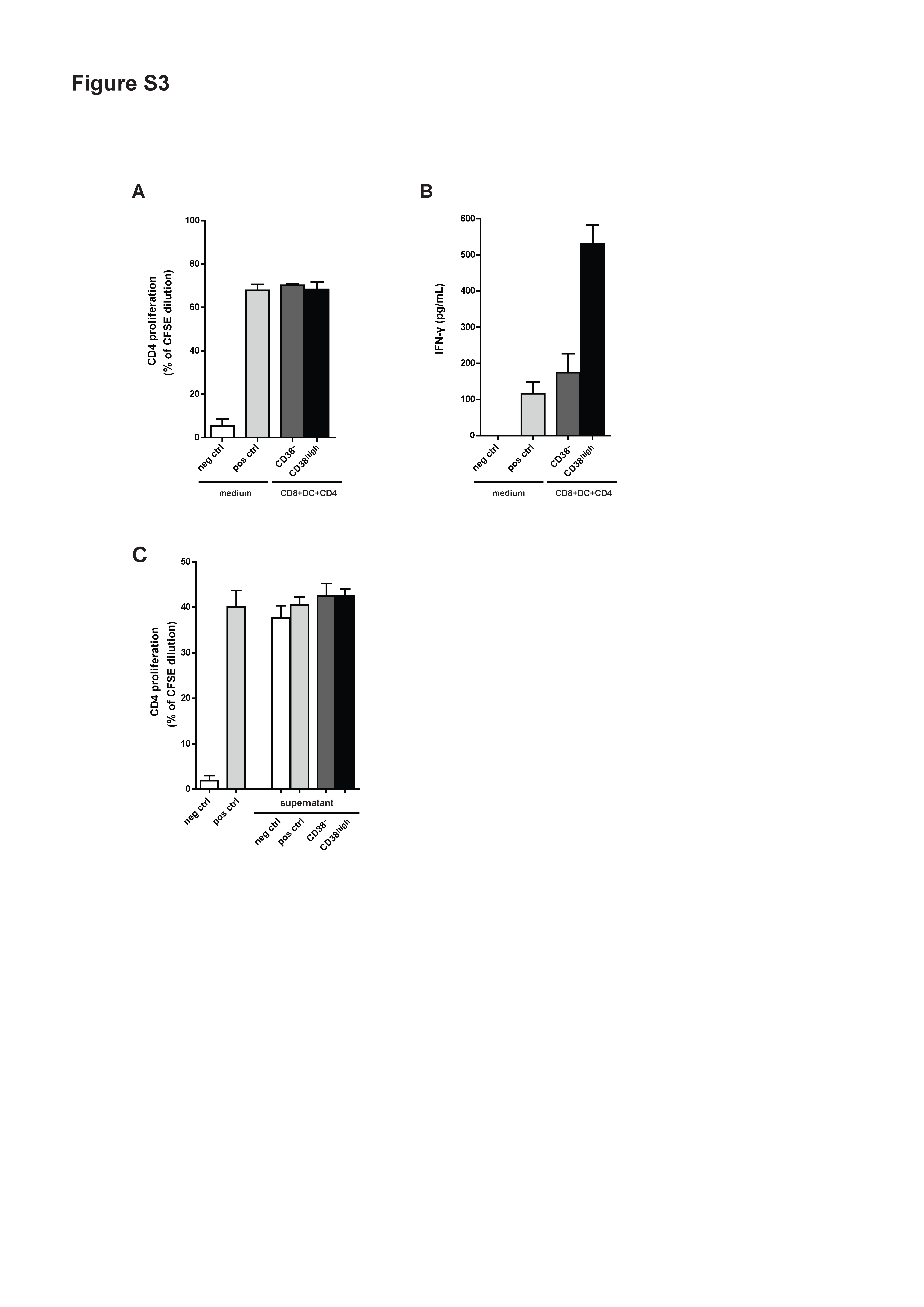

Supplement: Figure S3 — CD8+CD38high T cell mediated-suppression is dependent on cell-to-cell contact. (A-B) CD4+ OTII T cells and DCs with (pos ctrl) or without (neg ctrl) OTII323–339 peptide were placed in the lower chamber of the 0.4µm transwell (Corning). Medium (medium) or indicated CD8+ T cells at a CD8:CD4 ratio of 1, CD4+ OTII T cells, DC and OTII323–339 peptide (CD8+DC+CD4) were given into the upper chamber of the transwell. (A) Antigen-specific proliferation of CD4+ OTII T cells from the lower chamber was measured by CFSE dilution after four days. (B) IFN-γ concentration in supernatants was measured by ELISA. Shown is the mean±SEM. (C) CD4+ OTII T cells were stimulated with the OTII323–339 peptide in the presence of DCs (pos ctrl). Supernatants collected from cultures with either CD4+ T cells and DCs (neg ctrl), CD4+ T cells, DCs and OTII323–339 peptide (pos ctrl) or CD4+ T cells, DCs, OTII323–339 peptide and either CD8+CD38− (CD38−) or CD8+CD38high (CD38high) T cells were added to the above described co-cultures at a dilution 1∶2. CD4+ OTII T cell proliferation was measured by CFSE dilution after three days of incubation. Shown is the mean±SEM of one representative experiment from two performed. (TIF) [file pone.0045234.s003.tif]

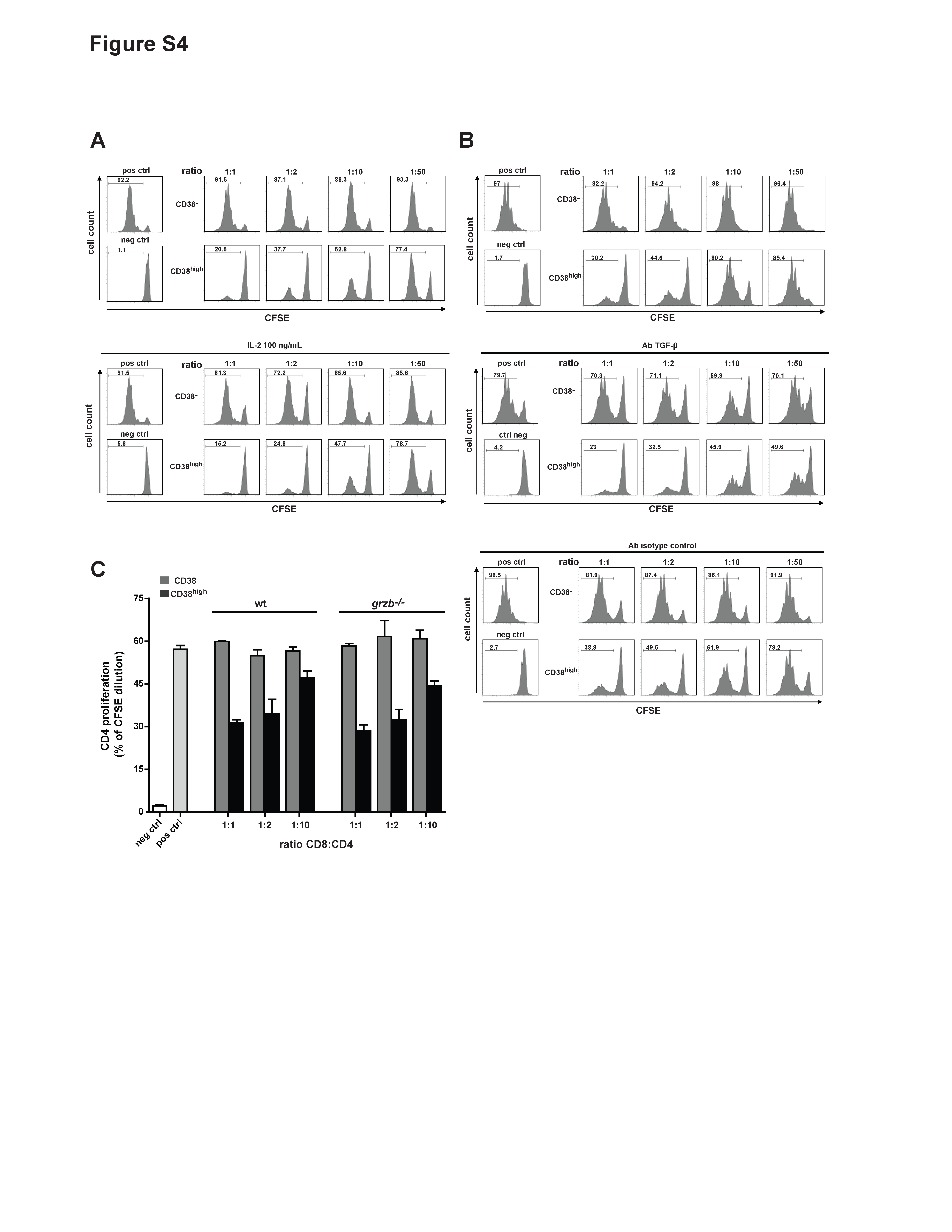

Supplement: Figure S4 — Mechanisms of CD8+CD38high T cell-mediated suppression of effector CD4 + T cell proliferation. CD4+ OTII T cells were used as responder and stimulated with OTII323–339 peptide in the presence of DCs. Different ratios of CD8+CD38− (CD38−) or CD8+CD38high (CD38high) T cells were added to the culture and proliferation of CD4+ OTII T cells was measured. Histograms show the CFSE dilution of the CD4+ OTII T cells after three to four days of culture (gated on CD4+ T cells). Numbers represent the percentage of proliferating cells. Pos ctrl represents the proliferation of CD4+ T cells incubated with DC and OTII323–339 peptide (without addition of CD8+ T cells). neg ctrl represents the proliferation of CD4+ OTII T cells incubated with DCs only (A-C). (A) IL-2 (100 ng/mL), (B) TGF-β Abs or isotype control Abs were added to the co-cultures or left untreated. (C) CD8+CD38high and CD8+CD38− T cells were sorted from C57Bl/6j wt or grzb−/− mice. The proliferation of CD4+ OTII T cells was measured by CFSE dilution after four days of culture. Shown is the mean±SEM of one of two independent experiments performed. (TIF) [file pone.0045234.s004.tif]
